# Supplementary material for: Phlogopite-Reinforced Natural Rubber (NR)/Ethylene-Propylene-Diene Monomer Rubber (EPDM) Composites with Aminosilane Compatibilizer
Source: Polymers (Basel). 2021 Jul 14;13(14):2318. doi: 10.3390/polym13142318 (PMC8309489; doi:10.3390/polym13142318)
Supplement: Supplementary file 1 [file polymers-13-02318-s001.zip › polymers-1266147-supplementary.pdf]

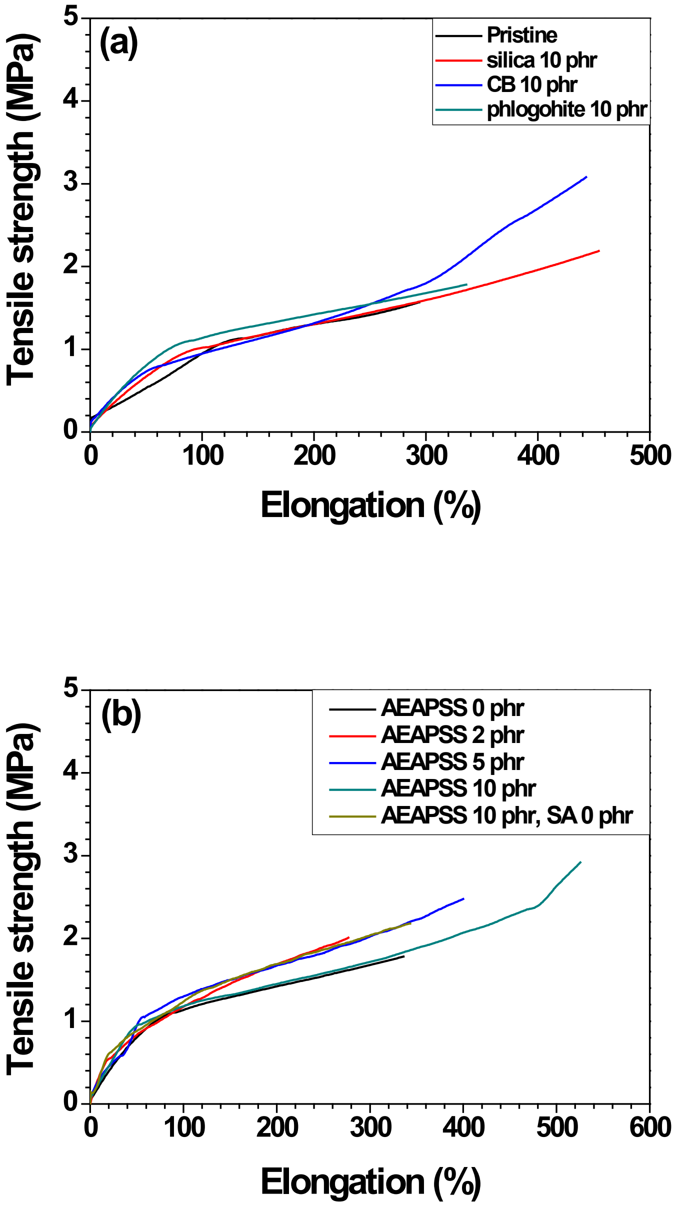

Figure S1. Stress-strain curves of NR/EPDM blends and composites with different fillers and AEAPSS concentrations: (a) Different fillers and (b) different AEAPSS concentrations with and without SA.

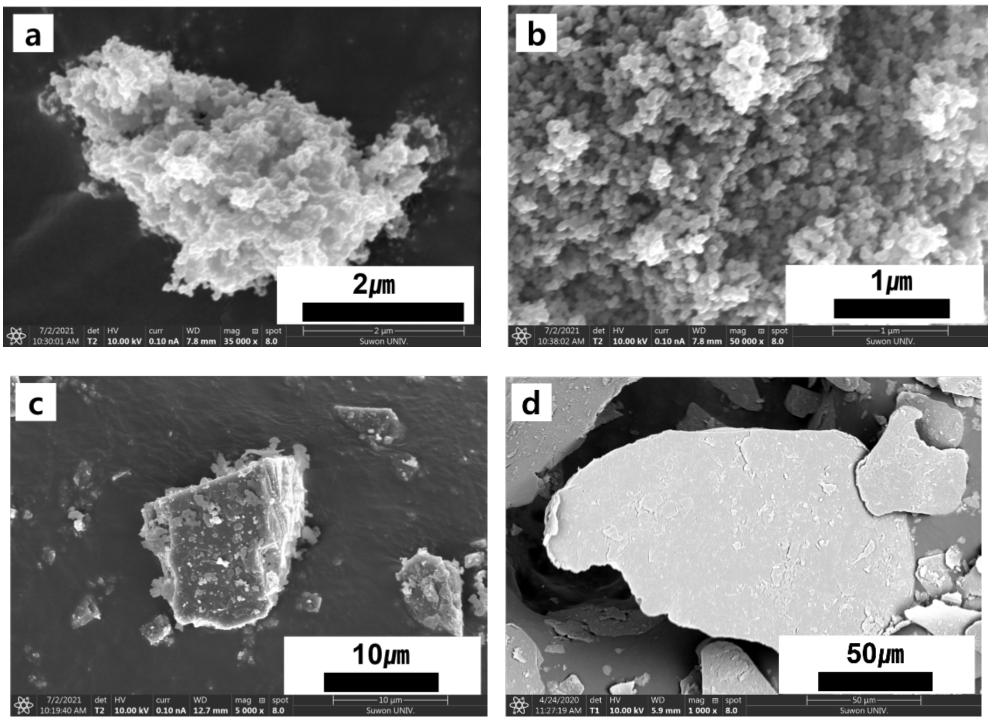

Figure S2. SEM images of pristine fillers: (a, b) CB, (c) silica, and (d) phlogopite

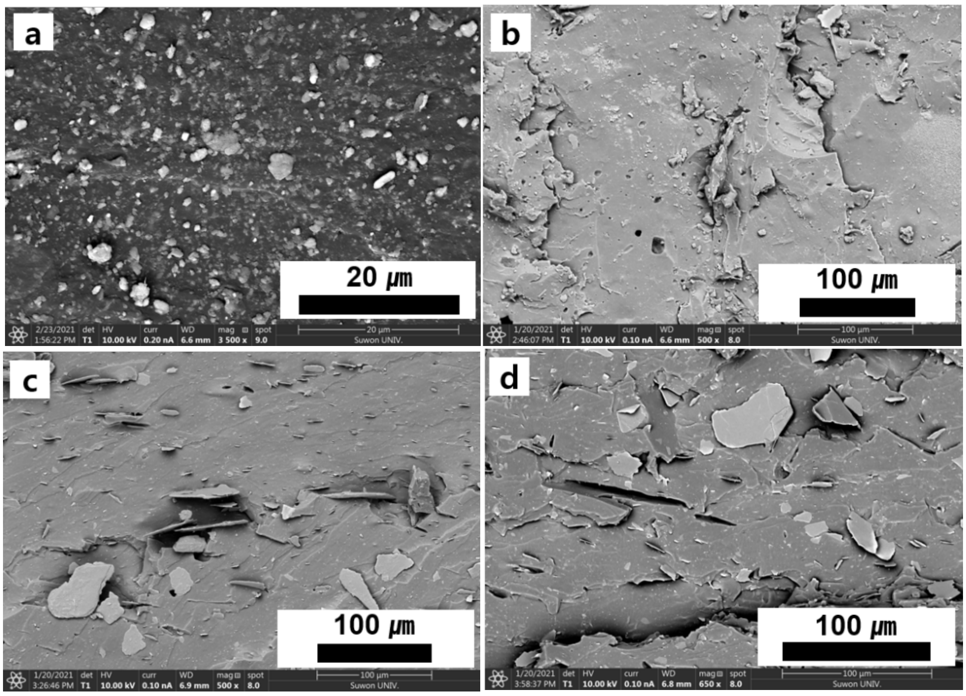

Figure S3. SEM images of the NR/EPDM composites with different fillers and AEAPSS concentrations: (a) 10 phr CB, (b) 10 phr silica, (c) 10 phr phlogopite, and (d) 10 phr phlogopite/10 phr AEAPSS
